# Effects of Sevoflurane on the Proliferation, Migration, and Xenograft Growth of HepG2 Hepatocellular Carcinoma Cells: An Exploratory In Vitro and In Vivo Study †

**DOI:** 10.3390/medicina62071267

**Published:** 2026-06-30

**Authors:** Kyong Sik Kim, Yeojung Kim, Keuna Shin, Aung Soe Paing, Sujin Baek, Boohwi Hong, Chaeseong Lim

**Affiliations:** 1 Department of Anesthesiology and Pain Medicine, Chungnam National University College of Medicine, Daejeon 35015, Republic of Korea; david2430@cnuh.co.kr (K.S.K.); yeojung80@cnuh.co.kr (Y.K.); iamsuza@cnuh.co.kr (S.B.); 2 Department of Anesthesiology and Pain Medicine, Chungnam National University Sejong Hospital, Sejong 30099, Republic of Korea; 3 Research Institute for Medical Sciences, Chungnam National University, Daejeon 35015, Republic of Korea; keuna@cnu.ac.kr; 4 Department of Surgery, 1000-Bedded Naypyitaw General Hospital, Naypyitaw 15011, Myanmar; aungsoepaing@gmail.com; 5 Department of Anesthesiology and Pain Medicine, Chungnam National University Hospital, Daejeon 35015, Republic of Korea

**Keywords:** anesthesia, sevoflurane, hepatocellular carcinoma, HepG2, cell migration, cell proliferation, xenograft

## Abstract

*Background and Objectives*: Sevoflurane, a widely used inhalational anesthetic, is frequently administered during hepatocellular carcinoma (HCC) surgery, including hepatic resection and orthotopic liver transplantation. Because such procedures often require prolonged anesthetic exposure, the potential influence of sevoflurane on HCC cell behavior is of clinical interest. We aimed to evaluate the effects of sevoflurane on the proliferation and migration of HepG2 cells in vitro and on tumor growth in a xenograft mouse model in vivo, and to explore whether hypoxia-inducible factor-1α (HIF-1α) might be involved in this process. *Materials and Methods*: For the in vitro experiments, HepG2 cells were exposed to room air (0%), 2%, or 4% sevoflurane. A scratch wound healing assay was used to assess cell migration, and the number of viable cells was quantified by hemocytometer counting on day 4 to estimate proliferation. For the in vivo experiments, BALB/c nude mice bearing HepG2 xenografts were exposed to room air, 2% sevoflurane, or 4% sevoflurane for 3 h, three times weekly for 5 weeks. Tumor size and tumor weight were measured at the end of the exposure period. HIF-1α protein levels in tumor tissue were measured by enzyme-linked immunosorbent assay (ELISA) in tumor lysates and normalized to total tumor protein as an exploratory mechanistic analysis. Given the small sample available for this endpoint, the analysis had limited sensitivity to detect modest differences. *Results*: When wound closure was quantified and pooled across the analyzable experiments, no statistically significant difference was detected among the room air, 2% sevoflurane, and 4% sevoflurane groups (day-2 closure 19.9 ± 32.1%, 22.1 ± 25.8%, and 22.3 ± 28.8%, respectively; repeated-measures ANOVA *p* = 0.82), with variability dominated by between-experiment rather than treatment differences. In the proliferation assay, the number of viable HepG2 cells on day 4 was significantly lower in the 2% sevoflurane group (62.6 ± 3.3 × 10^5^) than in the room air group (68.5 ± 4.2 × 10^5^; *p* < 0.05); the 4% sevoflurane group (66.0 ± 3.2 × 10^5^) showed an intermediate value that did not reach statistical significance. In the xenograft model, mean tumor size in the room air, 2% sevoflurane, and 4% sevoflurane groups was 7.1 ± 1.9, 2.7 ± 2.0, and 2.1 ± 0.9 cm^3^, respectively (*p* = 0.041 for room air vs. 2% sevoflurane; *p* = 0.034 for room air vs. 4% sevoflurane). Tumor weight was likewise lower in the sevoflurane groups (room air, 7.88 ± 2.2 g; 2% sevoflurane, 2.95 ± 2.1 g; 4% sevoflurane, 2.3 ± 1.6 g; *p* = 0.044 for room air vs. 2% sevoflurane; *p* = 0.067 for room air vs. 4% sevoflurane). No statistically significant differences in tumor HIF-1α protein levels were observed among the three groups. *Conclusions*: In this exploratory study, sevoflurane exposure was associated with reduced HepG2 xenograft tumor growth in vivo, whereas its in vitro effects were more limited: a reduction in viable cell number was observed only at 2% sevoflurane, and an effect on cell migration could not be confirmed when analyzed across experiments. Tumor HIF-1α levels did not differ significantly between groups, suggesting that other molecular pathways may be involved. Further mechanistic and clinical studies are warranted before any conclusions can be drawn about the relevance of these findings to the perioperative management of patients with HCC.

## 1. Introduction

Hepatocellular carcinoma (HCC), the most prevalent type of liver cancer, is the fifth most common malignancy and the second leading cause of cancer-related mortality among Korean men in 2023 [1]. Despite advances in treatment modalities—including transarterial chemoembolization, radiofrequency or microwave ablation, percutaneous ethanol injection, cryoablation, radiation therapy, systemic chemotherapy, and molecularly targeted therapies—orthotopic liver transplantation and surgical resection remain the most effective curative options for HCC [2]. In South Korea in particular, living-donor liver transplantation for HCC has increased markedly in recent years [3]. Given that anesthesia is required for both hepatic resection and liver transplantation, anesthesiologists may need to consider whether the choice of anesthetic agent could influence short- and long-term oncologic outcomes.

Sevoflurane is one of the most widely used volatile anesthetics for the maintenance of general anesthesia. Its potential effects on tumor cell behavior have attracted increasing attention, but reported results are inconsistent across tumor types. Hirai et al. showed that sevoflurane can either stimulate or suppress cell proliferation, apoptosis, and invasion depending on the cancer cell line examined [4]. Sevoflurane has been reported to increase the growth, migration, or metastatic potential of glioma stem cells, glioblastoma cells, and renal cell carcinoma cells [5,6,7], whereas in colorectal cancer cells it induces apoptosis [8]. In lung cancer cells, sevoflurane exposure has been shown to decrease metastasis and invasion through modulation of hypoxia-inducible factor-1α (HIF-1α) and interactions with cancer-associated platelets [9,10]. Our group previously reported a dose-specific, rather than dose-dependent, suppressive effect of sevoflurane on A549 lung adenocarcinoma cells [11]. By contrast, evidence on the effects of sevoflurane on HCC cells is still limited.

We therefore hypothesized that clinically relevant concentrations of sevoflurane (2% and 4%) would inhibit the migration and proliferation of HepG2 cells in vitro and reduce HCC tumor growth in vivo. To test this hypothesis, we used a scratch wound healing assay to assess migration, a hemocytometer-based cell count assay to estimate proliferation, and a HepG2 xenograft mouse model to evaluate tumor size and weight. As an exploratory mechanistic step, we additionally measured tumor HIF-1α protein levels by enzyme-linked immunosorbent assay (ELISA), motivated by previous reports implicating HIF-1α in the effect of sevoflurane on other tumor cell lines [5,9]. Preliminary in vitro findings of this study were presented in abstract form at the ANESTHESIOLOGY 2023 Annual Meeting of the American Society of Anesthesiologists [12].

## 2. Materials and Methods

### 2.1. In Vitro Study

#### 2.1.1. Cell Preparation and Culture

HepG2 cells were obtained from the Korea Cell Line Bank (Seoul, Republic of Korea). Cells were cultured in high-glucose Dulbecco’s modified Eagle’s medium (Gibco, Grand Island, NY, USA) supplemented with 10% fetal bovine serum (HyClone, Logan, UT, USA), 100 U/mL penicillin, and 100 µg/mL streptomycin, and maintained at 37 °C in a humidified atmosphere containing 5% CO_2_. Culture medium was renewed every 48 h throughout the experimental period, including during the days of sevoflurane exposure.

#### 2.1.2. Sevoflurane Exposure System and Gas Monitoring

HepG2 cells were exposed to sevoflurane in a custom-designed airtight incubation chamber maintained at 37 °C with 5% CO_2_ and equipped with gas inlet and outlet ports. Sevoflurane was delivered from a calibrated vaporizer (Abbott Laboratories, Maidenhead, UK) through a closed gas delivery system with a calibrated air flow meter set to a constant total flow of 1 L/min. The outlet gas was sampled continuously and analyzed with a multigas analyzer (Datex Instrumentarium Corporation, Helsinki, Finland) to confirm that the target inspired sevoflurane concentration (0%, 2%, or 4%) was reached and maintained within ±0.2% throughout each exposure session. The chamber was pre-equilibrated for at least 5 min before the addition of cell culture plates. Exposures were thus performed under continuous-flow (dynamic) rather than static conditions. Culture plates were placed in the chamber with their lids ajar to allow gas equilibration, and fresh culture medium was provided at the start of each daily exposure to limit any influence of sevoflurane evaporation or adsorption to the plasticware. The 2% and 4% concentrations were selected to represent the lower and upper bounds of the clinically used range (approximately 1–2 minimum alveolar concentration) and to overlap with concentrations employed in comparable in vitro studies of volatile anesthetics in cancer cells.

#### 2.1.3. Scratch Wound Healing Assay (Cell Migration)

Cell migration was assessed using a scratch wound healing assay. HepG2 cells (5 × 10^6^) were seeded in 35 mm dishes and cultured to approximately 90% confluence. A single linear scratch was made across the cell monolayer of each dish with a 100 µL micropipette tip. The detached cells were removed by gentle washing with phosphate-buffered saline (PBS), and fresh culture medium was added. Cells were then exposed to room air, 2% sevoflurane, or 4% sevoflurane for 3 h daily on three consecutive days, using the chamber described in Section 2.1.2. Wound images were captured daily with an inverted microscope. The scratch assay was performed in five independent experiments. Because the experiments differed in seeding density, magnification, and manually generated baseline scratch width, wound closure was quantified post hoc from the archived images using an automated texture-based segmentation routine (custom script in Python version 3.14.6 [Python Software Foundation, Wilmington, DE, USA] with the scikit-image library version 0.26.0): for each image a local standard-deviation (texture) map was computed and thresholded relative to an Otsu cut-off, and the largest centrally located low-texture region was retained as the cell-free wound. The percentage wound closure for each condition within each experiment was then calculated as (A_0_ − A_t_)/A_0_ × 100, where A_0_ and A_t_ are the wound areas at day 0 and day t; this normalization removes between-experiment differences in magnification and absolute wound width. Three of the five experiments were imaged at a resolution adequate for reliable segmentation and were pooled for statistical analysis (Section 2.3). The remaining experiments, captured as low-resolution composite images at a near-confluent seeding density that did not permit reliable segmentation, were excluded from the quantitative analysis and are reported descriptively. Because each experiment provided one field per condition per time point, the experiment was treated as the unit of replication.

#### 2.1.4. Cell Proliferation Assay

**Figure 1:**
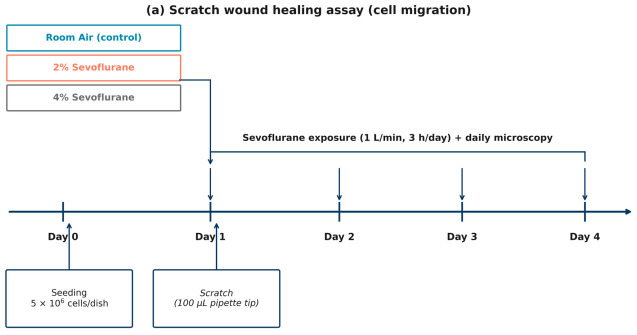

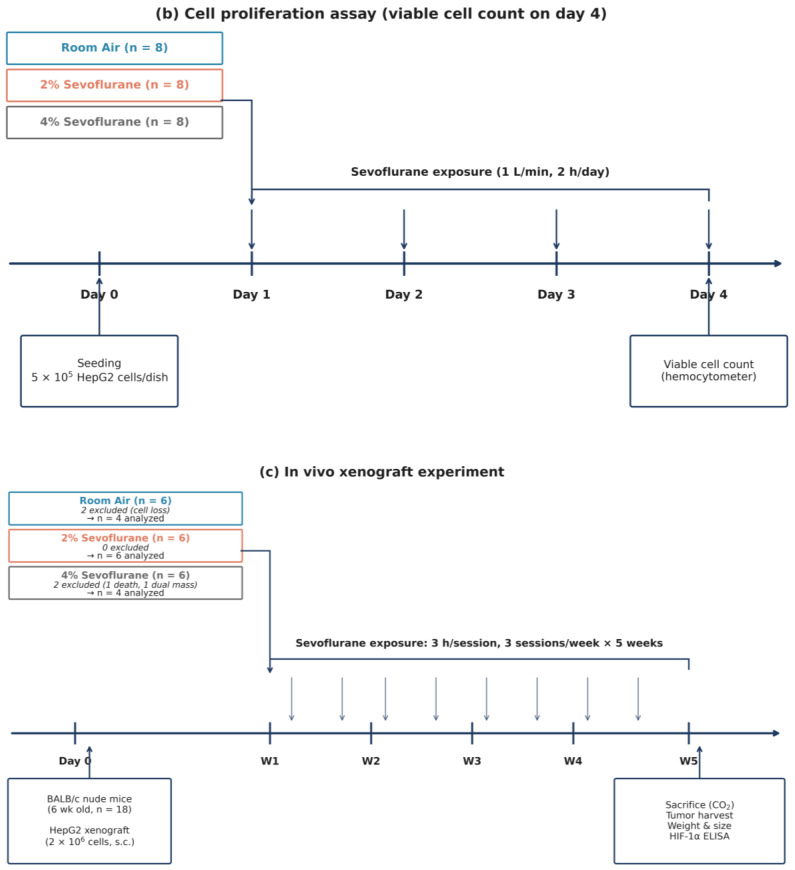
Schematic illustration of the experimental design. (**a**) Scratch wound healing assay: after scratching, HepG2 cells were exposed to 0%, 2%, or 4% sevoflurane for 3 h daily on three consecutive days. (**b**) Cell proliferation (viable cell count) assay: HepG2 cells were exposed to 0%, 2%, or 4% sevoflurane for 2 h daily on three consecutive days, and viable cells were counted on day 4. (**c**) In vivo study: HepG2 xenograft mice were exposed to room air, 2% sevoflurane, or 4% sevoflurane for 3 h three times weekly for 5 weeks.

On day 0, HepG2 cells (5 × 10^5^) were seeded in 24-well plates and 100 mm cell culture dishes (VWR International Ltd., Lutterworth, UK) and divided into three groups. Cells were cultured at 37 °C with 5% CO_2_ and exposed to 0%, 2%, or 4% sevoflurane for 2 h daily on three consecutive days (Figure 1b). Culture medium was refreshed every 48 h. On day 4, cells were detached with trypsin–EDTA, mixed with trypan blue (0.4%), and counted with a hemocytometer; only trypan blue–negative (viable) cells were included. Three high-power fields per well were also imaged for representative figures. As discussed below (Section 4), hemocytometer-based counting estimates the number of viable cells at a single time point and should therefore be interpreted as a surrogate measure of proliferation rather than as a direct quantification of proliferation kinetics or metabolic activity.

### 2.2. In Vivo Experiment

#### 2.2.1. Animal Preparation

Six-week-old male BALB/c nude mice (Damul Science, Daejeon, Republic of Korea) were acclimatized in our facility for 1 week before experimentation. Animals were housed under a 12 h light/dark cycle with 40–60% humidity at 20–26 °C and ad libitum access to food and water. All experimental protocols were approved by the Institutional Animal Care and Use Committee of Chungnam National University Hospital (approval number: 202112A-CNU-227; 5 January 2022; Daejeon, Republic of Korea) and conducted in accordance with the ARRIVE 2.0 guidelines.

#### 2.2.2. Xenograft Model and Anesthesia for Tumor Inoculation

To establish the xenograft model, each mouse received a subcutaneous injection into the right flank of 200 µL of PBS (pH 7.0) containing 2 × 10^6^ HepG2 cells. To minimize injection-related distress and to ensure consistent injection technique across animals, all mice in every group (including the control group) were briefly anesthetized with 3% sevoflurane for approximately 1 min immediately before injection. Because this very short induction exposure was identical across all groups, any potential anti-tumor effect of sevoflurane would be expected to apply equally to the controls and therefore to bias the comparison toward the null. We acknowledge that an alternative anesthetic regimen (for example, brief inhaled isoflurane or short-acting injectable agents) could have been used; this is discussed as a limitation in Section 4.

#### 2.2.3. Sevoflurane Exposure Protocol

Eighteen mice were randomly allocated by computer-generated random numbers into three groups (n = 6 each): room air (control), 2% sevoflurane, and 4% sevoflurane. The sample size was based on previous comparable HCC xenograft studies using inhaled anesthetics and on the practical considerations of this pilot experiment; a formal a priori power calculation was not performed, which is acknowledged as a limitation. Mice were placed in a transparent container submerged in a 38 °C water bath and exposed to 1 L/min air alone or to 2% or 4% sevoflurane mixed with 1 L/min air delivered through an animal anesthesia machine (R530SE; RWD Life Science Co., Ltd., Shenzhen, China). The outlet gas was again monitored with a multigas analyzer to confirm the target sevoflurane concentration. Exposures were performed for 3 h, three times weekly, for 5 weeks (Figure 1c).

#### 2.2.4. Measurement of Tumor Size and Body Weight

Tumor dimensions (length and width) and body weight were recorded three times weekly after each gas exposure. Tumor volume was calculated as tumor volume (cm^3^) = length × width^2^ × 0.5 [13,14].

#### 2.2.5. Tumor Excision and Weight Measurement

After 5 weeks of sevoflurane exposure, all mice were euthanized by CO_2_ inhalation in a sealed chamber, in accordance with American Veterinary Medical Association guidelines. Tumors were carefully resected, weighed, and dissected into individual tubes for further analysis.

#### 2.2.6. HIF-1α Measurement by ELISA (Exploratory Analysis)

Tumor tissue sections (2–3 cm) were rinsed with 0.7 mL of PBS to remove excess blood, weighed, and homogenized in 5–10 mL of ice-cold PBS using a glass homogenizer. The homogenate was further disrupted by ultrasonication three times on ice and centrifuged at 5000× *g* for 3 min; the resulting supernatant was used for ELISA. Total protein concentration of each supernatant was measured by the Bradford method, and supernatants were then diluted 1:10 in the assay diluent provided with a Human HIF-1α ELISA Kit (LSBio, Shirley, MA, USA) and assayed according to the manufacturer’s instructions. Briefly, 100 µL of diluted sample or standard was loaded into each well of a precoated microplate together with 100 µL of biotin-conjugated detection antibody and incubated for 1 h at 37 °C. After three washes, 100 µL of avidin–horseradish peroxidase conjugate was added and incubated for 30 min at 37 °C. After five washes, 90 µL of 3,3′,5,5′-tetramethylbenzidine substrate solution was added, the plate was incubated for 10–20 min at 37 °C in the dark, and the reaction was stopped by adding 50 µL of sulfuric acid stop solution. Optical density (OD) was read at 450 ± 2 nm, and HIF-1α concentrations were calculated from a standard curve generated with the recombinant standards supplied with the kit and normalized to the total protein content of each sample; values were therefore expressed in ng of HIF-1α per µg of total tumor protein (ng/µg protein). Because the in vivo experiment was conducted in two cycles and ELISA was performed on the samples from the second cycle only, the HIF-1α analysis was planned as an exploratory analysis. Specifically, HIF-1α was incorporated as an exploratory mechanistic endpoint only after the first xenograft cycle had been completed, so tumor lysates were available for this assay from the second cycle alone (*n* = 3, 3, and 2 for the room air, 2%, and 4% sevoflurane groups, respectively).

### 2.3. Statistical Analysis

Normality of the data was assessed using the Shapiro–Wilk test. Continuous variables are expressed as mean ± standard deviation. For comparisons among three groups, one-way analysis of variance (ANOVA) followed by Bonferroni post hoc tests was used for normally distributed data, and the Kruskal–Wallis test followed by Mann–Whitney U tests with Bonferroni correction was used for non-normally distributed data. Statistical analyses were performed with SPSS (version 26.0; IBM Corp., Armonk, NY, USA). A two-sided *p* value < 0.05 was considered statistically significant. Given the exploratory nature of this work and the relatively small number of animals per group, all *p* values should be interpreted cautiously. For the pooled scratch wound-healing analysis, the percentage wound closure on day 2 (the latest time point common to all analyzable experiments) was compared among the three conditions by repeated-measures ANOVA with experiment as a blocking factor, confirmed by the nonparametric Friedman test. Effect sizes are reported as partial η^2^ and as pairwise mean differences with 95% confidence intervals. For the xenograft endpoints, between-group differences in tumor volume and tumor weight were additionally summarized as pairwise mean differences with 95% confidence intervals and Hedges’ g and were re-examined by analysis of covariance (ANCOVA) with final body weight as a covariate to assess whether differences in tumor burden were independent of body weight.

During the preparation of this study, the authors used Claude (Claude Opus 4.8; Anthropic, San Francisco, CA, USA) for the statistical re-processing of experimental results that had not previously been usable for direct comparison because of minor differences in experimental conditions and procedures between runs. Specifically, the tool was used to assist in normalizing measurements within each independent experiment and pooling them across experiments with experiment included as a blocking factor (repeated-measures ANOVA, confirmed by the Friedman test), and in computing effect sizes (partial η^2^, Hedges g) with 95% confidence intervals and in the analysis of covariance adjusting for body weight. The tool was used solely for statistical computation; it was not used to generate, alter, or interpret the underlying experimental data. The authors have reviewed and edited the output and take full responsibility for the content of this publication.

## 3. Results

### 3.1. In Vitro Study

#### 3.1.1. Effect of Sevoflurane on HepG2 Cell Migration

**Figure 2:**
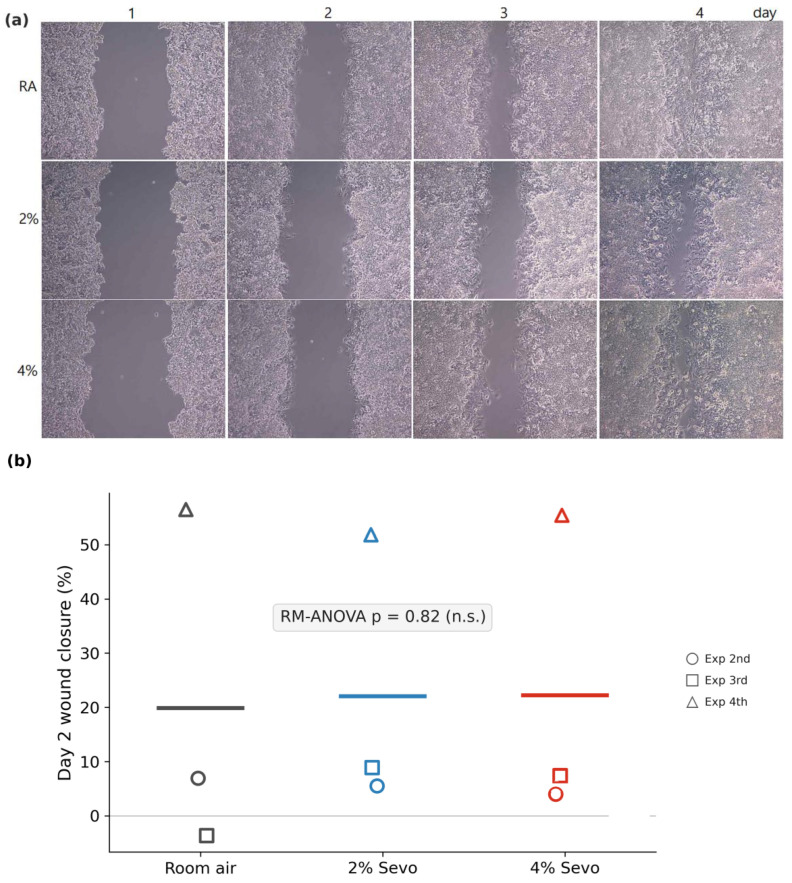
Effect of sevoflurane on HepG2 cell migration in the scratch wound healing assay. (**a**) Representative microscopic images of HepG2 cells exposed to room air (RA), 2% sevoflurane, or 4% sevoflurane on days 1–4 (×40). (**b**) Pooled wound closure across the three quantifiable independent experiments, expressed as the percentage reduction in wound area relative to day 0; each symbol represents one independent experiment and horizontal bars denote group means. No statistically significant difference was detected among the room air, 2% sevoflurane, and 4% sevoflurane groups (repeated-measures ANOVA *p* = 0.82; Friedman *p* = 0.72), and the variability was dominated by between-experiment differences (*p* = 0.0004). RA, room air; Sevo, sevoflurane.

To examine the effect of sevoflurane on HepG2 cell migration, we performed a scratch wound healing assay in five independent experiments. Because the experiments differed in seeding density, magnification, and baseline scratch width, wound closure was quantified as the percentage reduction in wound area relative to day 0 within each experiment, and the three experiments imaged at adequate resolution were pooled with experiment treated as a blocking factor. Pooled day-2 wound closure did not differ significantly among the room air, 2% sevoflurane, and 4% sevoflurane groups (19.9 ± 32.1%, 22.1 ± 25.8%, and 22.3 ± 28.8%, respectively; repeated-measures ANOVA *p* = 0.82, partial η^2^ = 0.09; Friedman *p* = 0.72), and the same was true at day 1 (*p* = 0.81). Pairwise differences between each sevoflurane concentration and room air were small, with 95% confidence intervals that included zero (2% vs. room air, +2.2%, 95% CI −20.5 to +24.9; 4% vs. room air, +2.3%, 95% CI −16.6 to +21.2). Variability was driven predominantly by a strong between-experiment effect (*p* = 0.0004) rather than by treatment (Figure 2). Thus, when analyzed across independent experiments, we did not detect a statistically significant effect of sevoflurane on HepG2 wound closure. The two experiments excluded for inadequate image resolution were set aside solely on the basis of image quality, independently of their results; on qualitative inspection they showed the same heterogeneous, between-experiment-dominated pattern, with no consistent direction of treatment effect, indicating that their exclusion did not bias the pooled analysis toward or away from the null.

#### 3.1.2. Effect of Sevoflurane on HepG2 Cell Numbers

**Figure 3:**
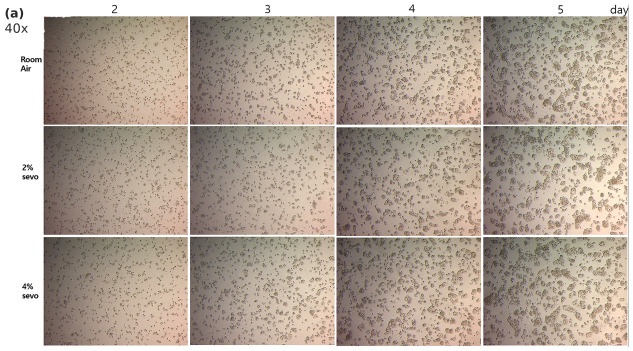

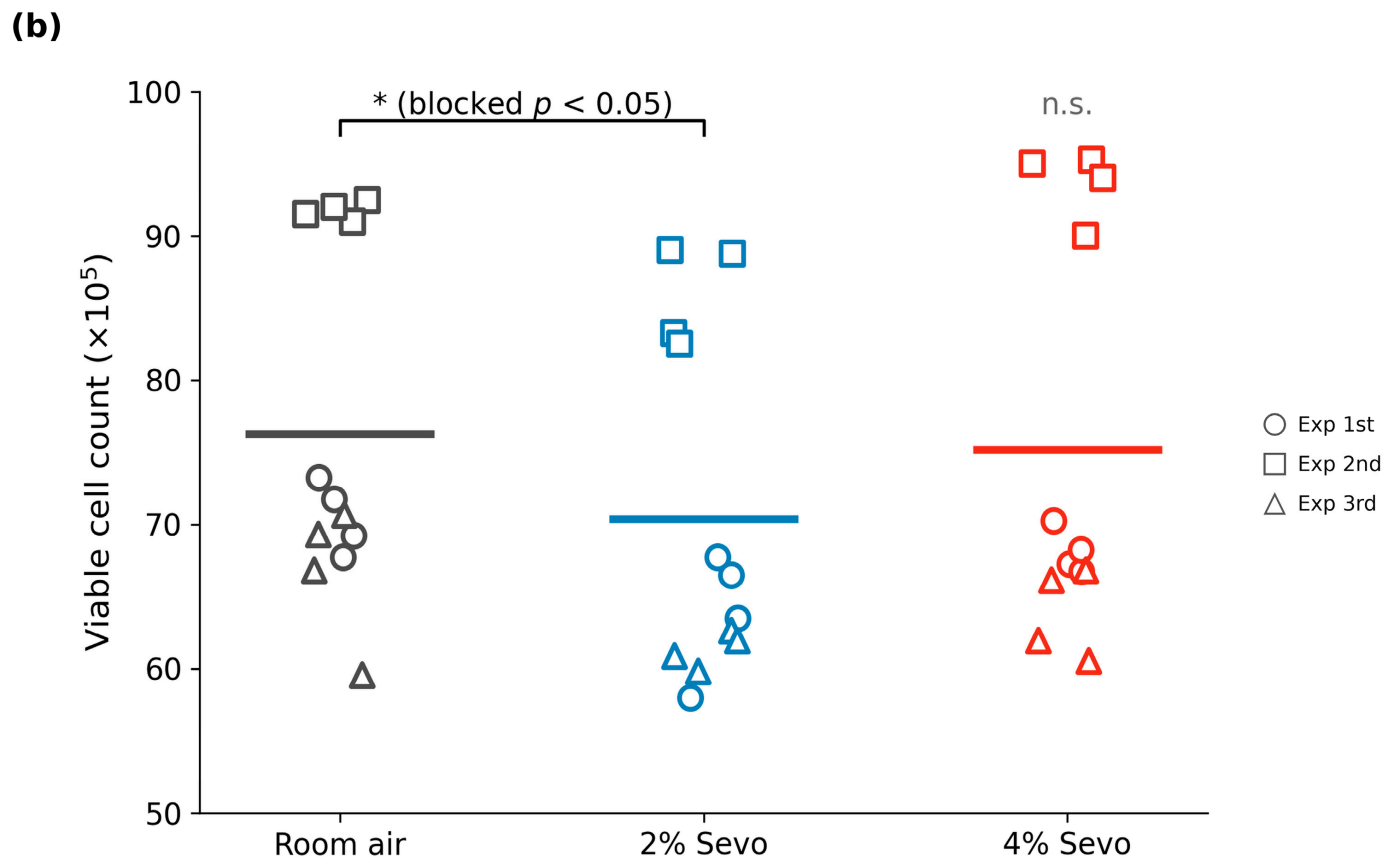
HepG2 viable cell numbers after sevoflurane exposure in vitro. (**a**) Representative microscopic images of HepG2 cells exposed to 0%, 2%, or 4% sevoflurane on days 2–5 (×40). (**b**) Viable HepG2 cell counts on day 4 across the three groups, pooled from three independent experiments (four dishes per group in each; each symbol denotes one dish and the symbol shape denotes the experiment, with horizontal bars indicating group means). Data are expressed as mean ± standard deviation. The reduction with 2% sevoflurane relative to room air was statistically significant when analyzed with experiment as a blocking factor (* *p* < 0.05), whereas 4% sevoflurane did not differ significantly from room air. Sevo, sevoflurane.

We next assessed the effect of sevoflurane on HepG2 cell numbers as a surrogate of in vitro proliferation. HepG2 cells were exposed to room air, 2% sevoflurane, or 4% sevoflurane and counted on day 4. The number of viable HepG2 cells was significantly lower in the 2% sevoflurane group (62.6 ± 3.3 × 10^5^) than in the room air group (68.5 ± 4.2 × 10^5^; *p* < 0.05). The 4% sevoflurane group showed an intermediate value (66.0 ± 3.2 × 10^5^) that did not differ significantly from either of the other two groups (Figure 3). This assay was performed in three independent experiments (four dishes per group in each). The reduction with 2% sevoflurane relative to room air was consistent across all three experiments (mean difference approximately −5.9 × 10^5^) and remained statistically significant when the experiments were pooled with experiment as a blocking factor (room air vs. 2% sevoflurane, *p* < 0.05; Hedges g = −1.6; 95% confidence interval of the difference −9.96 to −1.87 × 10^5^). By contrast, the 4% sevoflurane group did not differ significantly from room air (mean difference −2.6 × 10^5^, 95% confidence interval −6.6 to +1.5; Hedges g = −0.7), and the direction of its effect was inconsistent between experiments (Figure 3).

### 3.2. In Vivo Study

#### 3.2.1. Tumor Size and Weight in the Xenograft Model

**Figure 4:**
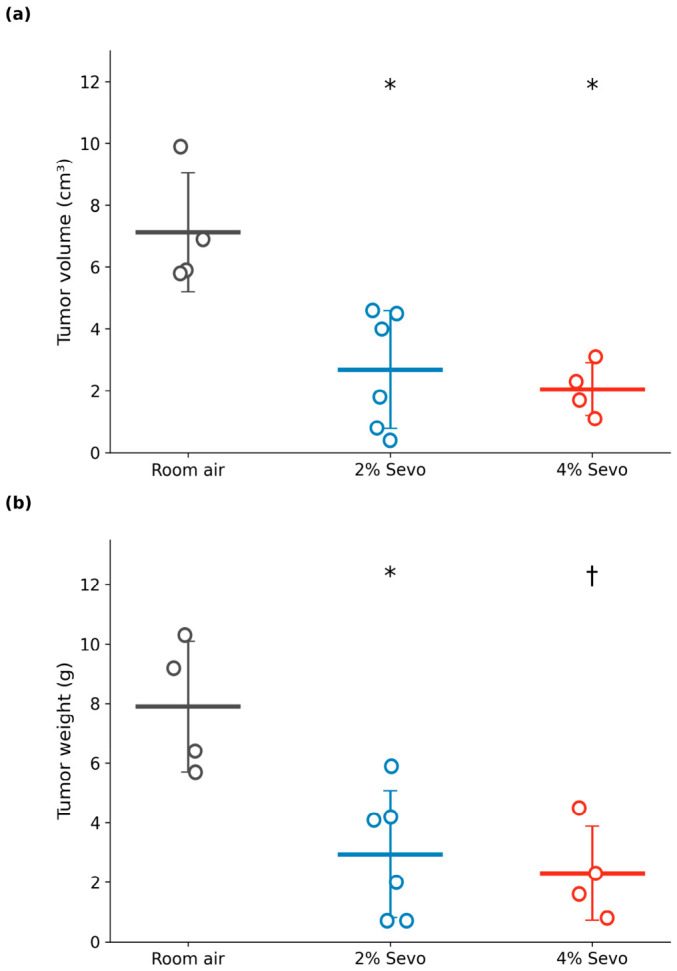

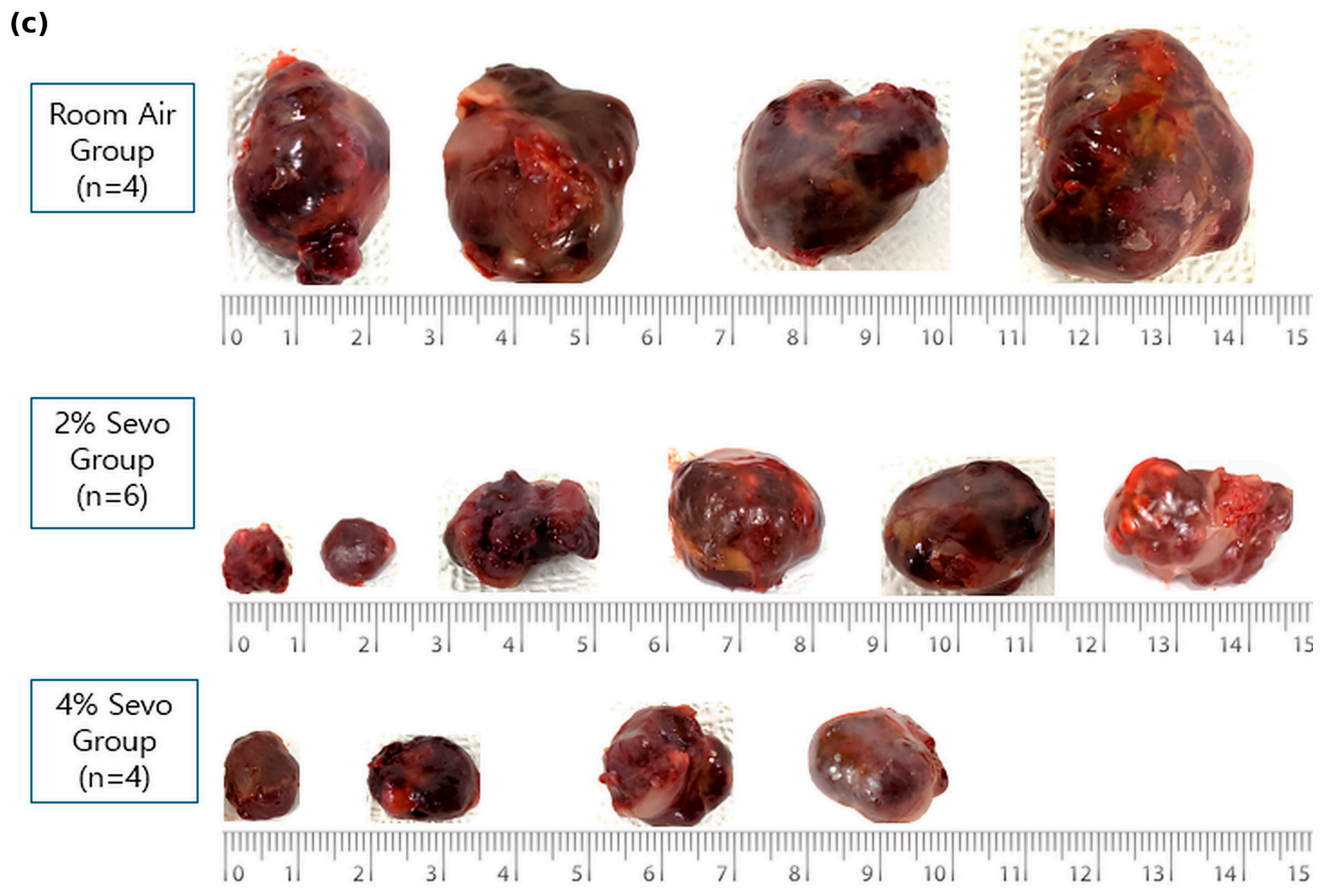
Comparison of HepG2 xenograft tumors among the three groups. (**a**) Tumor volume and (**b**) tumor weight. Each symbol represents one animal, and horizontal bars indicate group means ± standard deviations. * *p* < 0.05 vs. room air; † *p* = 0.067 for room air vs. 4% sevoflurane (tumor weight). (**c**) Photographs of all harvested tumors arranged by group. Per-animal data for the 14 xenograft mice included in the analysis are shown in Table 1. Sevo, sevoflurane.

Eighteen mice were randomly allocated equally to the room air, 2% sevoflurane, and 4% sevoflurane groups. Four mice were excluded for technical reasons (two in the room air group, both with HepG2 cell spillage at injection; two in the 4% sevoflurane group, one premature death and one with two separate masses at the injection site). Tumor formation was observed in all remaining animals at approximately 5 weeks after HepG2 cell injection, and tumor size increased progressively over time. Initial body weights did not differ between the room air (25.3 ± 0.8 g), 2% sevoflurane (24.4 ± 1.4 g), and 4% sevoflurane (24.3 ± 0.8 g) groups (*p* = 0.297). Final body weight and weight change differed significantly across groups (*p* < 0.05): the room air group had a higher final body weight (28.3 ± 3.1 g) than the 2% sevoflurane (22.7 ± 1.2 g) and 4% sevoflurane (25.0 ± 1.2 g; *p* = 0.012) groups, and a greater weight gain (2.97 ± 3.2 g) than the 2% (1.69 ± 1.88 g) and 4% (0.7 ± 1.0 g; *p* = 0.024) sevoflurane groups (Table 1). Mean tumor volume was 7.1 ± 1.9 cm^3^ in the room air group, 2.7 ± 2.0 cm^3^ in the 2% sevoflurane group, and 2.1 ± 0.9 cm^3^ in the 4% sevoflurane group (*p* = 0.041 for room air vs. 2% sevoflurane; *p* = 0.034 for room air vs. 4% sevoflurane; Figure 4a). Mean tumor weight was 7.88 ± 2.2 g, 2.95 ± 2.1 g, and 2.3 ± 1.6 g, respectively (*p* = 0.044 for room air vs. 2% sevoflurane; *p* = 0.067 for room air vs. 4% sevoflurane; Figure 4b). The omnibus comparison was significant for both endpoints (tumor volume, one-way ANOVA *p* = 0.002 and Kruskal–Wallis *p* = 0.018; tumor weight, *p* = 0.004 and 0.027), with large effect sizes. Relative to room air, tumor volume was lower by 4.44 cm^3^ (95% CI 1.48 to 7.40) with 2% sevoflurane and by 5.08 cm^3^ (95% CI 2.20 to 7.95) with 4% sevoflurane (Hedges’ g = −2.3 and −3.4), and tumor weight was lower by 4.97 g (95% CI 1.59 to 8.35) and 5.60 g (95% CI 2.20 to 9.00), respectively (Hedges’ g = −2.3 and −2.9). When final body weight was included as a covariate, the group difference remained significant for tumor volume (ANCOVA *p* = 0.044; body-weight covariate *p* = 0.52) and was of borderline significance for tumor weight (*p* = 0.058), indicating that the reduction in tumor burden was not explained by the lower body weight of the sevoflurane-exposed animals. On gross inspection, tumors were visibly larger in the room air group than in either sevoflurane group (Figure 4c).

#### 3.2.2. Tumor HIF-1α Protein Levels (Exploratory Analysis)

**Figure 5:**
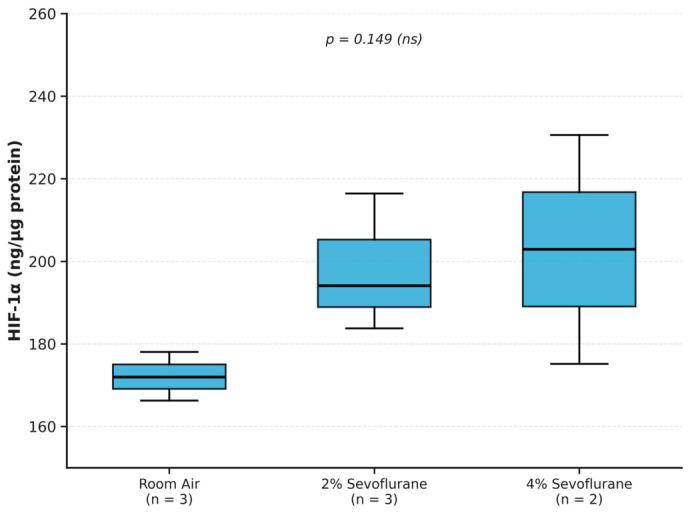
Tumor hypoxia-inducible factor-1α (HIF-1α) protein levels measured by ELISA, normalized to total tumor protein and expressed in ng/µg protein. Data are shown as box plots (median, interquartile range, and whiskers). The in vivo experiment was performed in two cycles, and ELISA was performed only on the samples from the second cycle, so the number of samples in this exploratory analysis is smaller than in Figure 4. No statistically significant difference was observed among the three groups (*p* = 0.149). Sevo, sevoflurane.

As an exploratory analysis, HIF-1α protein levels in tumor tissue were measured by ELISA and normalized to total tumor protein. Mean tumor HIF-1α concentrations were 172.1 ± 5.9 ng/µg protein in the room air group, 198.1 ± 16.7 ng/µg protein in the 2% sevoflurane group, and 202.9 ± 39.2 ng/µg protein in the 4% sevoflurane group, with no statistically significant difference among the three groups (*p* = 0.149; *n* = 3, 3, and 2 for the room air, 2%, and 4% sevoflurane groups, respectively; Figure 5). These results suggest that, in this model, the observed reduction in HepG2 xenograft growth is not accompanied by a measurable change in tumor HIF-1α protein levels, although the small sample size precludes any firm conclusion.

## 4. Discussion

General anesthesia is required for both hepatic resection and orthotopic liver transplantation—the two most effective curative treatments for HCC. In South Korea, the scarcity of deceased donors has driven a recent increase in living-donor liver transplantation for HCC [4]. Because anesthetic agents may interact with tumor biology, identifying agents with potentially favorable oncologic profiles is of increasing interest. Sevoflurane, a widely used volatile anesthetic, has favorable pharmacokinetics and a well-established safety profile, and preconditioning with sevoflurane has been associated with improved early graft function after pediatric living-donor liver transplantation [15].

In the present study, we evaluated the effects of clinically relevant concentrations of sevoflurane (2% and 4%) on HepG2 cells using a scratch wound healing assay and a hemocytometer-based cell count assay in vitro, and a HepG2 xenograft model in vivo. When wound closure was quantified and pooled across the three analyzable independent scratch assay experiments, no statistically significant difference was detected among the room air and sevoflurane groups, and the variability was dominated by between-experiment rather than treatment differences; we therefore did not find evidence that sevoflurane alters HepG2 cell migration under these conditions. In the cell count assay, the number of viable HepG2 cells on day 4 was significantly lower in the 2% sevoflurane group than in the room air group, consistent with a reduced viable cell number, which may reflect decreased proliferation, reduced cell survival, or other cellular processes; the 4% sevoflurane group showed an intermediate, non-significant effect. We emphasize, however, that hemocytometer-based viable cell counting reflects the net number of cells at a single time point and is influenced by proliferation, cell death, and adhesion; it is therefore a surrogate, rather than a direct, measure of proliferation [16]. Similarly, the scratch assay measures collective cell movement into a denuded area and does not distinguish migration from cell shape change or proliferation within the wound. These methodological constraints should be kept in mind when interpreting our in vitro findings.

Our findings are broadly consistent with previous reports indicating that sevoflurane can suppress migration in some cancer cell lines. In esophageal squamous cell carcinoma, a 10 h sevoflurane exposure suppressed migration, whereas no effect was seen in esophageal adenocarcinoma [17]. Conversely, a dose-independent promotion of breast cancer cell migration has been reported [18]. The reported effects of sevoflurane on cancer cell proliferation are similarly heterogeneous: a study exposing eight cancer cell lines to 1% sevoflurane found increased proliferation in six lines and decreased proliferation in the remaining two [5]. More directly relevant to our work, Zhu et al. showed that exposure of HepG2 and SMMC7721 HCC cells to 1.7%, 3.5%, and 5.1% sevoflurane for 6 h suppressed their migration and invasion in a dose-dependent manner, an effect attributed to downregulation of miR-665-mediated activation of the ERK/MMP pathway [19]. In contrast to that dose-dependent suppression, however, our pooled scratch analysis did not reveal a statistically significant migratory effect of sevoflurane on HepG2 cells; the slower closure noted in individual experiments was not reproduced once all analyzable experiments were combined, underscoring the importance of pooled rather than representative analysis and the possibility that differences in exposure duration, concentration range, and cell line handling contribute to the divergent findings across studies.

In the xenograft model, both 2% and 4% sevoflurane were associated with significantly smaller and lighter tumors compared with room air, but no significant difference was observed between the two sevoflurane concentrations. Previous xenograft studies have reported variable results for sevoflurane, including tumor growth suppression in SKOV3 ovarian xenografts via the JNK/p38 MAPK pathways [20], suppression of U251 glioma xenografts via upregulation of miR-218-5p [21], and, in contrast, increased growth and invasion of U87 glioblastoma xenografts associated with upregulation of CD44 [7]. Notably, in our model, 4% sevoflurane suppressed xenograft tumor growth despite not producing a statistically significant reduction in HepG2 cell counts in vitro. As discussed by Silliman and Wang [22], well-designed in vitro systems often fail to reproduce the complexity of the in vivo microenvironment, including stromal, immune, and vascular interactions and systemic pharmacokinetics, which may at least partially explain this discrepancy.

Hypoxia and the HIF pathway play an established role in HCC progression. Approximately 50–60% of rapidly growing solid tumors contain hypoxic regions, in which the HIF pathway promotes angiogenesis, metastasis, proliferation, and treatment resistance [23,24]. Several studies have identified elevated HIF-1α expression as a marker of poor prognosis and an indicator of vascular invasion and metastasis in HCC [25]. Liang et al. showed that 2.5% and 3.5% sevoflurane inhibited hypoxia-induced proliferation, migration, and metastasis of A549 lung cancer cells via inhibition of HIF-1α and downregulation of XIAP, survivin, fascin, and heparanase [10], whereas Shi et al. reported that sevoflurane promoted glioma stem cell expansion under normoxia in part through induction of HIF-1α and HIF-2α [6]. Motivated by these reports, we measured HIF-1α protein in tumor tissue as an exploratory mechanistic analysis. We did not detect a statistically significant difference in tumor HIF-1α levels among the three groups. This result should be interpreted with caution: the assay was performed on a subset of animals corresponding to the second of two experimental cycles, so the sample size for the HIF-1α analysis is smaller than for the size and weight comparisons. Taken at face value, however, our data suggest that in the HepG2 xenograft model the anti-tumor effect of sevoflurane is not primarily mediated by changes in steady-state HIF-1α protein levels, and that other pathways—such as the ERK/MMP axis implicated in HepG2 cells [19], or miR-mediated signaling described in other tumor types [21]—may be more relevant in this model. Further mechanistic studies, including assessment of additional hypoxia-related and proliferation-related markers, are needed. Given the absence of a significant difference and the small sample available for this endpoint, the HIF-1α analysis should be regarded as exploratory and hypothesis-generating only, and no mechanistic conclusion regarding HIF-1α can be drawn from these data.

This study has several important limitations. First, viable cell counting with a hemocytometer is less sensitive and less standardized than colorimetric proliferation assays such as MTT, MTS, or CCK-8, or BrdU/EdU incorporation assays, and provides only a snapshot of cell number rather than a direct measure of proliferation kinetics [16]. Replication of the present results with a dedicated proliferation assay is warranted. Second, the scratch assay assesses collective cell motility and cannot separate migration from morphological change or local proliferation; complementary methods such as Transwell migration assays would strengthen future work. In addition, the wound-healing experiments were limited by a single microscopic field per condition per time point, by imaging of non-identical fields across days rather than registered time-lapse, and by heterogeneity in seeding density and magnification between experiments; together with the small number of experiments suitable for quantification (*n* = 3), these factors left the pooled analysis underpowered to detect small migratory effects and may account for the large between-experiment variability observed. Furthermore, because wound closure was quantified post hoc from archived images during the revision rather than from data acquired prospectively for that purpose, this retrospective image analysis carries the limitations inherent to post hoc processing; to limit observer bias, segmentation relied on an automatic, data-driven threshold (the Otsu method, computed objectively from each image rather than chosen by the investigator) applied uniformly to all images without manual adjustment, although these parameters were not pre-specified before the original experiments. Third, all animals, including controls, received a brief (~1 min) induction with 3% sevoflurane at the time of HepG2 cell inoculation; although this exposure was identical across groups and is therefore expected to bias the comparison toward the null, we cannot fully exclude an effect on tumor engraftment. Fourth, our sample size was based on prior comparable xenograft work rather than on a formal a priori power calculation, and four animals were excluded for technical reasons, leaving small per-group numbers that limit statistical power, particularly for the HIF-1α analysis. Fifth, the use of HepG2 cells, which were originally derived from a hepatoblastoma rather than from a classical adult HCC [26,27,28], limits the generalizability of our findings to HCC; replication in cell lines with more typical HCC features, such as Huh7, would be valuable. Finally, tumor volumes measured with calipers may include surrounding tissue and may overestimate true tumor volume; we therefore weighed excised tumors as a complementary endpoint [14].

## 5. Conclusions

In this exploratory in vitro and in vivo study, both 2% and 4% sevoflurane were associated with reduced tumor size and weight in a HepG2 xenograft mouse model, whereas the in vitro effects were more limited: a reduction in viable HepG2 cell number was observed only with 2% sevoflurane, and an effect on cell migration could not be confirmed when analyzed across independent experiments. Tumor HIF-1α protein levels did not differ significantly between groups in this exploratory analysis, suggesting that the observed effect may involve molecular pathways other than HIF-1α. These findings should be regarded as hypothesis-generating; further mechanistic studies in additional HCC cell lines, replication using more sensitive proliferation and migration assays, and clinical investigations are needed before any inferences can be made about the perioperative management of patients undergoing HCC surgery or liver transplantation. In particular, given the small final per-group numbers remaining after the pre-specified animal exclusions and the absence of an a priori power calculation, the in vivo findings should be regarded as preliminary and require confirmation in larger, adequately powered studies.

## Figures and Tables

**Table 1:** Body weight of the 14 xenograft mice and weight of excised tumors.

	R1	R2	R3	R4	2S1	2S2	2S3	2S4	2S5	2S6	4S1	4S2	4S3	4S4
IW (g)	24.5	26.4	25.2	25.0	24.9	23.9	22.6	24.1	26.8	24.2	24.7	25.2	23.7	23.6
FW (g)	24.7	26.2	30.5	30.7	24.5	23.0	22.6	23.2	21.9	21.1	25.6	25.6	22.1	25.1
FTS (cm^3^)	5.9	6.9	9.9	5.8	0.8	4.6	4.5	1.8	4.0	0.4	2.3	1.1	1.7	3.1
STW (g)	5.7	9.2	10.3	6.4	0.7	4.1	5.9	2.0	4.2	0.7	2.3	0.8	1.6	4.5
SW (g)	24.9	26.4	30.9	30.8	24.0	23.6	22.5	23.3	22.2	20.9	25.6	25.6	23.2	25.7

R, room air; 2S, 2% sevoflurane; 4S, 4% sevoflurane; IW, initial body weight; FW, final body weight (measured on the last day of gas exposure); FTS, final tumor size (volume in cm^3^, calculated as length × width^2^ × 0.5); STW, sacrifice tumor weight (weight of the excised tumor); SW, sacrifice body weight (measured on the day after the final gas exposure, immediately before tumor excision following CO_2_ euthanasia).

## Data Availability

The raw data supporting the conclusions of this article will be made available by the authors on request.

## Abbreviations

The following abbreviations are used in this manuscript:

HCC	Hepatocellular carcinoma
HIF-1α	Hypoxia-inducible factor-1 alpha
ELISA	Enzyme-linked immunosorbent assay
PBS	Phosphate-buffered saline
ANOVA	Analysis of variance
ARRIVE	Animal Research: Reporting of In Vivo Experiments
